# “I was still very young”: agency, stigma and HIV care strategies at school, baseline results of a qualitative study among youth in rural Kenya and Uganda

**DOI:** 10.1002/jia2.25919

**Published:** 2022-07-12

**Authors:** Jason Johnson‐Peretz, Sarah Lebu, Cecilia Akatukwasa, Monica Getahun, Theodore Ruel, Joi Lee, James Ayieko, Florence Mwangwa, Lawrence Owino, Anjeline Onyango, Irene Maeri, Frederick Atwine, Edwin D. Charlebois, Elizabeth A. Bukusi, Moses R. Kamya, Diane V. Havlir, Carol S. Camlin

**Affiliations:** ^1^ Department of Obstetrics Gynecology, & Reproductive Sciences University of California San Francisco (UCSF) San Francisco California USA; ^2^ Infectious Diseases Research Collaboration (IDRC) Kampala Uganda; ^3^ Department of Pediatrics University of California San Francisco (UCSF) San Francisco California USA; ^4^ Kenya Medical Research Institute (KEMRI) Nairobi Kenya; ^5^ Department of Medicine University of California San Francisco (UCSF) Center for AIDS Prevention Studies San Francisco California USA; ^6^ Department of Medicine Makerere University College of Health Sciences Kampala Uganda; ^7^ Division of HIV Infectious Diseases & Global Medicine Department of Medicine University of California San Francisco (UCSF) San Francisco California USA

**Keywords:** adolescent, eastern Africa, highly active antiretroviral therapy, HIV, medication adherence, social stigma

## Abstract

**Introduction:**

Adolescents and young adults living with HIV (AYAH) have the lowest rates of retention in HIV care and antiretroviral therapy (ART) adherence, partly due to the demands of school associated with this life stage, to HIV‐related stigma and to fears of serostatus disclosure. We explore the implications of school‐based stigma and disclosure on the development of agency during a critical life stage in rural Kenya and Uganda.

**Methods:**

We conducted a qualitative study in the baseline year of the *SEARCH Youth* study, a combination intervention using a life‐stage approach among youth (15–24 years old) living with HIV in western Kenya and southwestern Uganda to improve viral load suppression and health outcomes. We conducted in‐depth, semi‐structured interviews in 2019 with three cohorts of purposively selected study participants (youth [*n* = 83], balanced for sex, life stage and HIV care status; recommended family members of youth [*n* = 33]; and providers [*n* = 20]). Inductive analysis exploring contextual factors affecting HIV care engagement revealed the high salience of schooling environments.

**Results:**

Stigma within school settings, elicited by non‐consensual serostatus disclosure, medication schedules and clinic appointments, exerts a constraining factor around which AYAH must navigate to identify and pursue opportunities available to them as young people. HIV status can affect cross‐generational support and cohort formation, as AYAH differ from non‐AYAH peers because of care‐related demands affecting schooling, exams and graduation. However, adolescents demonstrate a capacity to overcome anticipated stigma and protect themselves by selectively disclosing HIV status to trusted peers and caregivers, as they develop a sense of agency concomitant with this life stage. Older adolescents showed greater ability to seek out supportive relationships than younger ones who relied on adult caregivers to facilitate this support.

**Conclusions:**

School is a potential site of HIV stigma and also a setting for learning how to resist such stigma. School‐going adolescents should be supported to identify helpful peers and selectively disclose serostatus as they master decision making about when and where to take medications, and who should know. Stigma is avoided by fewer visits to the clinic; providers should consider longer refills, discreet packaging and long‐acting, injectable ART for students.

## 1 INTRODUCTION

*“It used to happen sometimes [seven years] back that I could forget about the appointment dates. I could even forget about where I keep my appointment card… I was still very young.”* (male aged 18, day‐scholar, Kenya)

Adolescents and young adults living with HIV (AYAH) face numerous challenges to engagement in the HIV care continuum: lower rates of retention in care and antiretroviral therapy (ART) adherence compared to adults [1, 2] and higher rates of virologic failure [3, 4]. The burden of HIV infections is disproportionately high among adolescents (between 10 and 19 years of age) and young adults (roughly between ages 18 and 24) living in low‐ and middle‐income countries. In Kenya, more than 51% of new HIV diagnoses in 2017 occurred among youth aged 15–24 years [5]. In Uganda, adolescents aged between 13 and 19 years account for 50% of people living with HIV nationally [1], with a mean ART adherence rate of 61–64% [1, 2].

Both AYAH and adults experience medication‐related burdens, such as drug side‐effects, fears or discomfort with size of pills, pill burden [6, 7] and stigma. Vulnerability to stigma, however, is often heightened during adolescence and may combine with other barriers to care engagement in this population [8, 9, 10, 11, 12, 13]. A study in Kenya reported that 57% of AYAH were lost to follow‐up and long‐term care efforts in part due to stigma in school settings [14]. In school settings, younger adolescents (aged 11–15) have limited agency over their living situations due to their dependence on caregivers [15]. In Uganda, AYAH may need to store and take medication under the supervision of school nurses, which can lead to involuntary disclosure [15]. Successful navigation of stigma and care engagement while in school has implications for later HIV care engagement. Transitioning out of school may further influence disengagement from care if not secured earlier in life [4, 16].

A minimalist reading of HIV stigma restricts it to “othering, blaming, and shaming,” to distinguish it from other forms of HIV discrimination [8, 17, 18, 19, 20, 21]. Crenshaw's theory of intersectionality expanded stigma analysis to “capture … contextual dynamics of power” beyond the lens of single‐axis analysis (e.g. gender alone and age alone) to include health status, disability, HIV status and age [21, 22, 23, 24, 25, 26].

Life‐course theory provides a useful framework for exploring how HIV stigma during adolescence operates as a constraining factor that can paradoxically promote the maturation of a sense of agency for AYAH during this life stage, as it necessitates the growth of skills for seeking HIV care opportunities in later life stages. Four principles structure life‐course analysis: the individual's geo‐historical context; the timing of transitions (e.g. from school to work‐life and parenthood) within that context; the interdependence of “linked lives” (e.g. social network development during youth sets up expectations about one's life‐course, relative to the life events of peers and prior generations); and human agency. Thus, life‐course theory posits that historical context and the timing of critical periods in life influence developmental trajectories and health outcomes.

Human agency is expressed by altering the timing of life events and choosing the path one will follow. “Individuals construct their own life‐course through the choices and actions they take within the opportunities and constraints of history and social circumstances” [9]. Agency is expressed within the synchronicity of life transitions in one's cohort of peers, as well as in the contingent opportunities, constraints and inter‐linked lives one is thrown into [27].

Using a life‐course lens, we examine the implications of school as a setting wherein agency is developed in the context of stigma and the opportunities afforded by education and changing generational relationships. We distinguish between younger and older adolescents to suggest that within the institutional setting of school, youth develop their sense of agency around medication adherence through support‐seeking and selective HIV disclosure (opportunity) and resistance to stigma (constraint). Agency in developing effective strategies to remain in care goes hand in hand with developing strategies to resist stigma. Both are learned in the school setting, and both entail making choices between opportunities and constraints [28, 29, 30].

The findings come from a longitudinal qualitative study examining the barriers and facilitators of HIV care engagement among youth enrolled in the “SEARCH Youth” study (Strategic Antiretroviral Therapy and HIV testing for Youth in Rural Africa, NCT03848728), a community cluster‐randomized controlled trial in 28 rural communities in Kenya and Southwestern Uganda (14 communities per country). The study included a life‐stage counselling component for youth, drawing on insights from life‐course theory [9].

School‐going AYAH experience amplified challenges to care engagement because school is a place of intense scrutiny from peers and adults where any difference is set in sharp relief [31]. During the transition from childhood to adulthood, young people tend to value acceptance and appreciation from their peers. In their absence, social marginalization occurs, which can be amplified by stigma [15]. HIV‐related stigma in school environments can be increased by limited privacy, lack of family support and inadequate systems for supporting students living with HIV and their engagement in care [32, 33, 34, 35, 36, 37]. For young people, disclosing one's HIV (positive) status can be associated with loss of friends, bullying, social exclusion and denied schooling [34, 38]. Yet, lack of disclosure has itself been associated with poorer health outcomes [18]. Understanding age‐related constraints and opportunities, therefore, not only provides insight into how AYAH grapple with HIV‐related stigma, but can also inform future interventions which address AYAH‐identified barriers to adherence.

## 2 METHODS

### 2.1 Study context

“SEARCH Youth” is a combination‐intervention study designed to address structural barriers and psychosocial needs through life‐stage adapted counselling, technology‐enabled provider mini‐collaboratives, rapid viral load feedback and structured‐choice clinic access, aimed at improving viral suppression among AYAH aged 15–24 years.

Through in‐depth, semi‐structured interviews, a qualitative component aims to identify barriers, facilitators and mechanisms of action of the study intervention at the patient, family, clinic and community (school and village) levels. This article presents findings from baseline data collected prior to intervention.

### 2.2 Participants and procedures

The qualitative study used in‐depth, semi‐structured interviews with (1) a youth cohort of 83 participants of young people aged 15–24 years, purposively selected from 8 of the 28 communities in SEARCH‐Youth study (balanced by study arm and region), with participant selection balanced by HIV care status (newly diagnosed; out of care for the past 6 months; and engaged in care), sex and age; (2) a cohort of 33 family members of the youth cohort, purposively selected upon recommendation from youth participants about which family member they trusted and was most involved in their HIV care; and (3) a provider cohort (*n* = 20) purposively selected from two intervention clinics per region to include clinical officers, nurses and peer educators. The three cohorts permitted triangulation of data on barriers and facilitators of HIV care. We did not interview school personnel who were not part of the formal clinical study. For this paper, we define younger adolescents as between 15 and 17 years, older adolescents as 18–20 years and those aged 21–24 as young adults.

### 2.3 Data collection

A gender‐balanced team (three men and three women) of trained qualitative researchers, native speakers of the local languages, administered the interviews at baseline between June and December 2019. Confidentiality was ensured by conducting interviews in private rooms in clinic facilities or community locations convenient for the participants. Audio recordings were transcribed and translated into English.

Interviews with AYAHs explored types and breadth of social support systems, experiences of HIV care and HIV status disclosure. Family member interviews explored perceived attitudes of schoolmates and others towards AYAHs, HIV care and treatment of AYAHs, and perceived burden of caregiving to AYAHs. Provider interview guides included perspectives on the needs and barriers to *care engagement* among AYAHs, and perceived challenges to *care delivery* to AYAH. The purpose of the qualitative interviews was not exclusively focused on school‐based HIV stigma; rather, what emerged during our analysis was school as a site of stigma as well as support in resisting such stigma in order to engage in HIV care during a particular life stage. Reporting this emergent finding is useful for shaping future interventions in this demographic.

### 2.4 Data analysis

A six‐person team transcribed and coded data using Dedoose software (LO, AO, CA, FA, IM and JL]. An initial coding framework with broad codes was defined on the basis of topic areas from theory‐informed interview guides developed by the project lead (CSC). The larger team (including MG and CSC) reviewed and discussed an initial set of transcripts and difficult coding segments, refined the broad codes with child codes and yielded a final coding framework reflective of both *a priori* concepts from theory and empirical research, and emergent codes reflecting phenomena and concepts derived from inductive coding of the data [39]. Codebook definitions and examples of code applications were reviewed throughout the analysis period. Our research question was: what are the barriers and facilitators to HIV care engagement common to youth in both Uganda and Kenya?

We used a thematic analysis on the resulting coded excerpts [40, 41]. Thematic analysis revealed the high salience of school settings as a context which alternately supports and undermines ART adherence for study participants currently or recently in school. We then undertook a deeper analysis of transcript excerpts via re‐coding for terms related to the emergent theme of school contexts—principal, teacher(s), student(s), exam(s) and class(es). Subgroup analyses from this recoding were restricted to age, rather than gender and location, because of the limits of the data set we were using.

### 2.5 Author(s) position statement(s)

The team‐based analytical approach was enriched by the lived experience—“the knowledge gained by an individual through direct encounter with a phenomena” [42, 43]—of research team authors who had first‐hand experience of school environments in the study setting (SL and others). Research team members who gathered data in Dholuo (LO and AO) and Runyankole (CA and FA) also engaged in data analysis and confirmed interpretations of the data to which additional team members contributed (JJP, MG and CSC). This ensured that the translation and interpretation of the data respectfully captured the nuances of participants’ voices [44]. The researchers acknowledge that some may disagree with our interpretations on the basis of their differing experiences of the same environments.

### 2.6 Ethical approval

The University of California San Francisco Committee on Human Research, the Ethical Review Committee of the Kenya Medical Research Institute, the Makerere University School of Medicine Research and Ethics Committee and the Uganda National Council of Science and Technology all approved this study as minimal risk for all participants, including minors. All study participants provided written informed consent to participate in the study.

## 3 RESULTS

Participants in the youth cohort of the parent clinical study were aged 15–24 years and presented a mix of school‐going (18%) and non‐school‐going (82%) individuals (including those who had recently left school). Youth in the qualitative sample reflected the composition of the SEARCH‐Youth study population, achieving balance by region, treatment status and sex. The composition of the family member cohort, in contrast, was driven by youth cohort members’ selection preferences, and was predominantly female (60%), with more partners (51.5%) than parents (24.2%) (Table 1).

**Table 1:** Characteristics of study participants, by interview cohort

Cohort type	Characteristic	*n* (%)
Youth cohort	Sex	
	Female	54 (65.1%)
	Male	29 (34.9%)
	Age	
	15–17 (Younger adolescent)	16 (19.3%)
	18–20 (Older adolescent)	22 (26.5%)
	21–22 (Early young adult)	22 (26.5%)
	23–25 (Late young adult)	23 (27.7%)
	Care status (baseline)	
	New to care	34 (41.0%)
	Already in care	39 (47.0%)
	Re‐engaging in care	10 (12.0%)
	Region	
	Uganda	38 (45.8%)
	Kenya	45 (54.2%)
Healthcare workers’ cohort	Sex	
	Female	12 (60.0%)
	Male	8 (40.0%)
	Region	
	Uganda	12 (60.0%)
	Kenya	8 (40.0%)
Family cohort	Sex	
	Female	20 (60.6%)
	Male	13 (39.4%)
	Relationship	
	Partner	17 (51.5%)
	Parent/caregiver	8 (24.2%)
	Other	8 (24.2%)
	Region	
	Uganda	14 (42.4%)
	Kenya	19 (57.6%)

School‐going individuals included day‐scholars and boarding school students. This paper focuses on them because tension between education and care engagement emerged as a key theme in our analysis. Stigma and disclosure decisions repeatedly emerged as affecting ART adherence and clinic attendance, and informed relationships with peers and older adults. Below, we present findings supported by excerpts from the transcripts and roughly aligned with the four principles of life‐stage analysis: geo‐historical context and timing of life‐stage transitions; linked lives between peers and cross‐generationally; and agency illustrated through choices within constraints and opportunities at school.

### 3.1 Education in the context of community and life stage

Most participants lived with at least one parent, a sibling or another member of the family. Participants reported living within a 1‐hour walking distance of their primary HIV care clinic. Overall, Ugandan participants were mostly engaged in agriculture for subsistence or livelihood, while in Kenya, most male participants were engaged in fishing, and women in running small businesses. School‐going youth may or may not end up in these occupations after schooling is completed. This vision of adulthood is a baseline expectation for the future, which schooling potentially expands beyond.

Both youth and caregivers recognized life‐stage changes parallel attending and exiting school and that education facilitates opportunities. Thus, although school may be a site of stigma and ART adherence obstacles, the rewards of school merit facing these challenges. One Ugandan grandmother was adamant children in her care receive an education, while two older adolescents in Kenya mentioned plans to continue onto college to become social workers or teachers. The increased opportunities afforded by education are illustrated by one participant as she makes her transition out of adolescence and into early adulthood:

*“As someone who has gone to school, I might get a job somewhere on a short notice …. As someone who has finished school, I tend to be very mobile.”* (female aged 19, Kenya)

As she indicates, life‐stage synchronization in later adolescence becomes more diversified as opportunities open up within historical and personal circumstances.

However, school opportunities are sometimes hindered by HIV care, setting AYAH students apart from their peers and normative expectations for schooling, exams and graduation. For younger adolescents, school schedules can work against medication and clinic times. As one participant observed going to clinic for refills on school days means missing parts of class *“that may have ended before you come back and … they can't rewind the already covered topic.”* (male aged 18, Kenya, day‐scholar). Choosing between class or medication remained particularly acute during exam periods and preparatory classes, which were explicitly cited as potential causes of medication interruption. Students were understandably unwilling to miss those particular classes.

Life‐stage theory notes development and its attendant choices are shaped by the interdependence of lives across generations. Our data confirm that sometimes an adolescent's life‐stage transition from school to work overlaps with a caregiver's own transition from work to retirement, which can impact both education and continuity in care:

*I: “Are you concerned of any possible future barrier to his drugs adherence?”*

*P: “I may not predict that. I am supposed to retire next year which may compromise his education because of the reduced income.”* (Uncle of male aged 18, Kenya, day‐scholar)

#### 3.1.1 Linked lives, disclosure and social support via parents, teachers and pupils

“*I talked to the head teacher as well as his deputy …The deputy head teacher stays at school with the children. He told [my grandson], ‘Don't you worry because even among us the teachers, there are some who are infected with HIV and we take our meds well because we want to have good health – so you should not be embarrassed about your situation.’ So when I talked to the teachers, he understood his situation better. The teachers also understood his situation and they made sure that he takes his meds well. He is now doing fine*.” (grandmother of a male aged 16, Uganda)

The older generation taking the lead for younger adolescents in navigating anticipated stigma also shows the interdependence of generations. Caretakers often brokered a supportive relationship with a school figure to help the student stay engaged in care—and in school. Several participants cited selective disclosure to at least one teacher who will always grant permission to visit the clinic as key in helping them stay engaged in care. In an interesting twist to students describing the importance of having teachers as allies and inspiration, a young teacher in the youth cohort was encouraged to stay adherent to ART on the day he tested positive when his cousin pointed out that he was not alone at school—even though he was a teacher:

“*He talked to me at length over the phone, reminding me of even many other people including pupils and students of the same HIV status. So, it was about being faithful to the drugs as the only option.”* (male aged 22, Kenya, new to care)

In this instance, the example of his students inspired a young‐adult teacher to remain adherent; the younger generation provided an example to a slightly older youth. What is interesting here is the difference in timing between when HIV infection was acquired, with most of the pupils acquiring it perinatally, but the teacher in young adulthood.

#### 3.1.2 Linked lives, otherness and belonging: peer groups

Cross‐generational links may help adolescents with institutional actors, but adolescents must demonstrate more agency when navigating peer‐to‐peer relationships. Here*, anticipated stigma* played a role in challenging care. Health education classes at school reportedly reduced enacted stigma, but not necessarily anticipated stigma. While one younger adolescent reported never experiencing stigma directly, and acknowledged that classmates educated about HIV would assist him during school classes when he fell ill, he “just feels it [enacted stigma] can happen” because he has seen other classmates stigmatized for HIV. In those cases, he observed, *“sometimes they [the perpetrators] are punished or suspended when the case reaches the administration.”* In such instances, the anticipation of stigma may paradoxically reinforce social support‐seeking behaviour.

Nevertheless, a sense of loneliness and self‐othering can persist among some older adolescents:

*“I really feel bad when I see people of my age living without the virus while I am the only one living with the virus.”* (male aged 18, Kenya, day‐scholar)

Joining a peer group with other AYAH, formally structured by a school or clinic, or informally when students unexpectedly see classmates at clinic and discover one another's status, facilitated care engagement. These networks reinforced commitment to medication schedules by ensuring students know they are not alone. A young male student (aged 15) observed of his AYAH peers, “*We usually motivate each other to adhere to the drugs well and also not to miss any appointments.”* This sense of belonging applied both to younger participants and older ones:

“*P: [The local clinic] is good, as I told you before; we do bookings for adolescents” day and low viral load day. When we meet as adolescents, we have a lot in common and we would not go gossiping about each other*.

*I: What happens on adolescents’ day?*

*P: We play together and encourage one another to adhere well*.

*I: Do you disclose your status to everyone who is in the adolescents’ group?*

“*P: Yes, we normally say that in this group we are all HIV positive and are all on HIV care and treatment as well. This makes them feel that it is a normal occurrence since even the providers are receiving HIV care and treatment.”* (female aged 19, Kenya)

This participant noted that the adolescent day not only relieved her of the fear of gossip, but it also normalized her status as living with HIV, which created a safety network where the adolescents encouraged one another to stay in care. This synchronicity may carry over into longer‐term support as the young people transition out of school.

#### 3.1.3 Agency and ART as an agent of disclosure, leading to stigma at school

“*We have people who are envious within our community and you might disclose to them – then they will mock you with it, and that will demoralize or discourage you.”* (female aged 19, Kenya)

Agency is shown within the constraints and opportunities of a historical context and social network. Stigma is one such social constraint for AYAH, especially when such stigma might end up discouraging ART adherence. Some participants mentioned experiencing stigma in the surrounding community in the form of gossip intended to tear others down. School does not necessarily negate community attitudes, since both students and teachers carry community attitudes with them. At school, students cannot easily avoid their peers.

Many participants expressed concern that taking medication during school hours could reveal their status and elicit a stigmatizing response. This fear was mentioned by providers, family and adolescents:

“*She shared that she is supposed to take her drugs at 8pm and this forces her to carry drugs to class, which she is not comfortable doing. They are usually given a five‐minute break and that is when she tries to fix her time with the drugs, which again is not working out so well because everybody shares the break and her friends do not equally want to leave her alone. She ends up missing doses because of that*.” Provider (Non‐facility based – Kenya)

“*I went to his school and I talked to his teacher. He told me that [my grandson] was afraid of taking his meds because he did not want his schoolmates to know that he was infected with HIV – that if they ever get to know, they would start to make fun of him.”* (grandmother of male aged 16, Uganda)

Anticipated stigma sometimes is not unfounded; participants also experienced enacted stigma. One student mentioned how his classmates make fun of his medication and the effect it had on him:

“*The children at school always say that I take big pills like those they give pigs. … I started to hate myself*.” (male aged 16, Uganda)

When such stigma is repeated, it runs the risk of discouraging students from continuing their medication schedules. In fact, taking medicine while at school involves several steps: getting the medicine into the school, finding a place to store it privately and take it soundlessly, excusing oneself to take it alone or to head to the nurse station and finding water or food to take with the medicine. One student reported using tap water that had not been boiled to take medicine while at school, so his classmates would not discover he was taking ART. Each of these points is an opportunity for one's status to be discovered by others; yet, identifying the available choices and pursuing some over others exhibits agency, and this agency develops as youth mature:

*When I was in school I was free with a teacher and one of the workers. We used to be inspected when getting into school so I would hide my pill in my bag and I would choose that specific person. I later thought that that if it was only one person who knew about it I would have a problem when they were not around, so I decided to disclose to some of the teachers and the deputy principal; my dad helped me disclose to them. The deputy said that the infection was normal and if I wanted to continue with my studies then I should. Some of the students would leave their medication at the nurse's office, but I told them that I would just manage on my own*.

*I: Do some of them give the school nurse their medication to keep for them?*

“*P: Yes, but the nurses may also share out your status to others. I refused and decided to keep them in my box under lock and key and would use them at my own time.”* (female aged 19, Kenya)

The above excerpt highlights how some students manage support from school faculty and staff, and the concern, clearly present but not shared by everyone, that nurses may be less trustworthy about student HIV status than faculty. Importantly, this participant was among the older adolescents, and was better able, with her father's help, to assert her own agency in taking the medication.

## 4 DISCUSSION

This qualitative study conducted among adolescents and young adults living with HIV, their family members and care providers in eastern African communities, has elucidated actions AYAH undertake to resist forms of HIV‐related stigma. The findings show how a refusal of stigma through selective disclosure to trusted allies, and inventing of ways to adhere to ART, leads to increased sense of agency and better health outcomes among AYAH during a key developmental life stage. This essential skill develops partly by resisting HIV stigma through selective disclosure to trusted teachers or staff, and through identifying and reaching out to helpful peers. Both older and younger adolescents stressed the importance of support systems at home and among staff at school, with older adolescents showing greater ability to direct these relationships and disclosures, and younger adolescents relying on parents or caregivers to pave the way. Earlier interventions to improve health outcomes for AYAH may have missed opportunities to leverage such life stage‐based approaches, which have the potential for greater impact on adolescents [45].

Enacted stigma caused some youth to drop out of school (though not in every case), whereas anticipated stigma did not. Anticipated stigma did tempt younger adolescents to drop out of school, but was managed through speaking to teachers and adults, as the quote from the grandmother in Uganda demonstrated. In fact, the weight of our evidence suggests that navigating anticipated stigma permitted agency to mature for school‐going youth, more than facing enacted stigma—perhaps because anticipated stigma permits extended reflection (often with trusted adults) and does not require immediate response. Adults could take the youth through a process of reflection on options for navigating around or coping with stigma, and tapping support systems. Being guided through such questions helps youth develop skills they will need later when confronting non‐HIV‐related challenges in life.

Stigma is, therefore, a rather immediate obstacle amenable to adolescent agency when other choices are still out of reach. For AYAH, taking ART paradoxically increases stigma (especially self‐stigma) [46]. Yet, during a time of critical life‐stage transitions and during the baseline year of an intervention study, youth were already engaged in finding ways to manage healthcare for themselves, while refusing to internalize stigma to become invested with greater responsibility for their own and their families’ health [47, 48, 49].

### 4.1 Limitations

Our cohort was a mix of boarding and day scholar informants, which may limit its generalizability to one or the other setting. However, baseline data do suggest our sample is fairly representative of AYAH in Kenyan and Ugandan schools, and similar to school‐age cohorts throughout Anglophone Africa. Our findings agree with previous studies that despite the optimism brought by a sense of agency, school intensifies stigma triggers, with boarding schools especially ideal for such triggers to flourish and interrupt ART adherence [35, 50, 51].

We focused on school as a site of stigma without comparison to village‐based stigma, partly because though challenging, school is a potential island of support. For this reason, we also did not focus on adolescents who exited school early. We did not interview school personnel as they are not part of the formal clinical study. Follow‐up research should specifically triangulate with this group (admin, teachers, school nurses and security).

We did not use explicit probes for how adolescents developed a sense of agency over ART adherence, or how that agency developed differently among male and female participants; identifying this theme lays the groundwork for probes in follow‐up interviews during this longitudinal study. Although we did not examine how adolescents identified trustworthy candidates for disclosure, we uncovered accounts of managing the process with caregiver assistance.

## 5 CONCLUSIONS

Several recommendations follow from the implications our findings have for the HIV care continuum. Our participants mostly acquired HIV perinatally and were linked to care early. Their status was disclosed to them when they were old enough to understand. Introducing younger adolescents to a peer group of other AYAHs (especially including schoolmates) at the time of disclosure may create an initial support framework for resisting stigma by showing younger adolescents they are not alone [18]. This also creates the linkages between peers, which promotes synchronicity in life‐course expectations and may help carry through adherence into adulthood, especially given that older adolescents expressed concern about managing their care once they graduated or found work away from family and school [29].

For older adolescents, their life stage is a key opportunity to establish successful engagement in care by appealing to their growing sense of (self‐) responsibility, especially within a context where stigma is a reality. Through modelling opportunities for selective disclosure to teachers, staff and peers, providers can counsel adolescents on the key life skill of building supportive social and professional networks, while facilitating retention in care as the adolescent becomes an adult. Supported selective disclosure also confronts anticipated stigma directly by building confidence and discernment of others’ character, as a concrete aspect of this skill. Securing at least one person from the administration or one teacher as an ally to easily excuse them from class with minimal additional explanation facilitated engagement in care.

The findings also highlight the importance of school settings as places where ART adherence is both challenged and supported. Stigma is avoided by fewer visits to the clinic, which means providers should consider longer refills and discreet packaging, or the rollout of long‐acting ART to students as a key population. Appropriate food and potable water availability at schools is important for taking ART; and addressable by the school staff when possible. Keeping medication on site helped somewhat with adherence, except when on‐site nurses are mistrusted because anticipated stigma raises confidentiality concerns. HIV education in school supported stigma reduction when teachers promptly responded to enacted stigma among students. Finally, teacher support for medication reminders or dorm room arrangements for boarders may also encourage adherence by decreasing opportunities for stigma to be enacted [51]. Interventions supporting agency around strategies AYAH already use to resist stigma and engage in care, in the context of care delivery cognizant of the constraints created by educational institutions, can support youth during this critical period of multiple life transitions.

## COMPETING INTERESTS

The authors declare they have no competing interests.

## AUTHORS’ CONTRIBUTIONS

JJP led analysis and wrote the manuscript, with contributions from CSC. SL drafted the background section. CA drafted the methods section. JA, FM, LO, FA, CA and AO as members of the core research team did initial analysis, data collection, curation and coding of the transcripts. MRK, TR and DVH conceived the parent study, revised critically and with CSC issued final approval. CSC conceived the original qualitative project, revised critically and issued final approval.

## FUNDING

Funding for this study was provided by the National Institutes of Health/National Institute of Child Health and Human Development, grant number UG3 HD96915‐01.

## Data Availability

Source data are transcribed and translated interview data, which require redaction prior to sharing to protect confidentiality, but are readily available upon request.
